# Genome Sequence of a Novel Iflavirus from mRNA Sequencing of the Pupa of *Bombyx mori* Inoculated with *Cordyceps militaris*

**DOI:** 10.1128/genomeA.01039-15

**Published:** 2015-09-17

**Authors:** Tomohiro Suzuki, Yoshino Takeshima, Toshiyuki Mikamoto, Jun-David Saeki, Tatsuya Kato, Enoch Y. Park, Hirokazu Kawagishi, Hideo Dohra

**Affiliations:** a Center for Bioscience Research and Education, Utsunomiya University, Utsunomiya, Tochigi, Japan; b Research Institute of Green Science and Technology, Shizuoka University, Suruga-ku, Shizuoka, Japan; c Department of Applied Biological Chemistry, Faculty of Agriculture, Shizuoka University, Suruga-ku, Shizuoka, Japan; d Nichihara Research & Development Laboratories, Inc., Tsuwano, Kanoashi, Shimane, Japan; e Graduate School of Science and Technology, Shizuoka University, Suruga-ku, Shizuoka, Japan

## Abstract

We discovered a novel iflavirus from the transcriptome of the *Bombyx mori* pupa inoculated with the insect-pathogenic fungus *Cordyceps militaris*. The assembled iflavirus genome has 10,119 nucleotides, with a 3′-polyadenylated tail, and it encodes a polyprotein composed of 3,004 amino acids.

## GENOME ANNOUNCEMENT

The domestic silkworm *Bombyx mori* is well studied and has been of interest due to its excellent characteristic as a textile fiber; recently, it has also been used as a host for the expression of eukaryotic proteins. In this study, the pupa of *B. mori* was used as a host of the insect-pathogenic fungus *Cordyceps militaris*. The RNA sequencing (RNA-seq) analysis of *B. mori* inoculated with *C. militaris* detected an iflavirus genome sequence. Iflaviruses are positive-sense single-stranded RNA viruses that infect insect hosts, such as moths and butterflies (*Lepidoptera*), honey bees and ants (*Hymenoptera*), and brown planthoppers and aphids (*Hemiptera*) (1–3). Several iflaviruses are known to have pathogenicity to their hosts, often leading to diarrhea, developmental malformations, and death of the host (2).

We sequenced the mRNA of *B. mori* (Kinsyu-Showa strain) (Nichihara Research & Development Laboratories, Inc.) inoculated with *C. militaris* (NBRC 100741). Total RNA was extracted from three male samples of *B. mori* using the TRIzol reagent (Life Technologies) and further purified using the RNeasy plant minikit (Qiagen). Strand-specific RNA sequencing libraries were prepared using the SureSelect strand-specific RNA library prep kit (Agilent Technologies) and sequenced using an Illumina MiSeq sequencer. In total, 11.7 million 76-bp paired-end reads were generated, of which 238,946 reads (2.05%) were derived from the iflavirus genome. The raw reads were cleaned up with cutadapt (4) by trimming adapter sequences and low-quality ends (quality score, <30), discarding reads <50 bp, and with the FASTX-Toolkit (5) by trimming the last 76 bases, resulting in 236,539 paired-reads totaling approximately 35.3 Mb. The cleaned reads were *de novo* assembled using Trinity (6). The assembled single contig has 10,119 nucleotides, with a G+C content of 38.7%, terminates in a 3′-polyadenylated tail, and shows an average 3,486× coverage of the total length of the iflavirus genome. As a result of the annotation by Prokka (7), the iflavirus genome was found to encode a polyprotein composed of 3,004 amino acids. The BLASTp search to the NCBInr protein database for the polyprotein showed 73% amino acid sequence identity with that of the gypsy moth *Lymantria dispar* iflavirus 1 (3), but it did not show such high similarity (23% identical) with that of the same host *B. mori* infectious flacherie virus (1). These results and the phylogenetic analysis (data not shown) of the amino acid sequences of the polyproteins suggest that the iflavirus is a novel species of the genus *Iflavirus*. The iflavirus was scarcely detected in hot-air-drying pupae, suggesting that the iflavirus genome was degraded at a high temperature.

Here, we report the genome sequence of a novel iflavirus detected from the pupa of *B. mori* inoculated with *C. militaris*. However, little is known about interactions between the iflavirus and *C. militaris* in a host pupa. It might be a good target for future studies to elucidate the effects of the iflavirus on developmental stages of *C. militaris*, such as infection to a pupa, reproduction, and fruiting body formation.

### Nucleotide sequence accession number.

The genome sequence has been deposited in DDBJ under the accession no. LC068762. The version described in this paper is the first version.
